# The pathogenesis of cataract in professional workers exposed to solar radiation in marine environment

**Published:** 2020

**Authors:** Mauro Salducci, Gianfranco Gioia

**Affiliations:** * Department of Sense Organs, Faculty of Medicine and Dentistry, Medical Legal Ophthalmology, Sapienza University of Rome, Italy

**Keywords:** cataract, professionally exposed workers, solar radiation

## Abstract

In this article we presented the results on the cataract obtained in patients recruited during a period of two years in the social and labor ophthalmology ambulatory of Sapienza University in Rome. The first of two homogeneous populations (regarding the number of patients, age, profession, constant exposure to sunlight, same living habitat, geographical areas with similar intrinsic and extrinsic risk factors) involved the areas of the gulf of Pozzuoli and the gulf of Olbia, both of which are located at 40.5-41 degrees north latitude on the sea. The first place is located on the European continent and the other one on a medium-large island with particular genetic factors. The second of the two homogeneous populations involved the island of Ischia (NA) and the maritime professional activities carried out in Villasimius (CA) and surroundings, respectively at 40- and 39-degrees north latitude on the sea. The first is a medium-small island and the second one is located on a medium-large island, but both are in the middle of the Mediterranean Sea and are characterized by a certain degree of genetic isolation.

## Introduction

The enrolment period of the current year was from the beginning of June to the end of July, with the maximum intensity of the solar radiation and also the major number of hours of radiation at our latitudes. We selected 2 homogeneous populations of port workers, nautical moorers, seafarers and lifeguards, who have been exposed for several years (at least 10) to the sun rays during the central hours of the day in the afore-mentioned period. The inclusion criteria were also a natural bilateral vision equal to or greater than 5/ 10, no past eye diseases and the sub-continuous use of protective sunglasses during their work having no graduated or specified construction technical characteristics [**1**]. The choice of the 2 geographical areas was dictated by the presence of the maximum solar radiation in summer, which is increased by the natural reverberation of sandy and marine surfaces, as widely reported in international literature [**2**,**3**]. Moreover, the density of the local population that is substantially autochthonous and sedentary and the relative lack of polluting industrial activities in these areas made it possible to verify whether or not there was a genetic predisposition for senile and pre-senile cataracts in the island populations considered [**4**].

## Materials and methods

The two populations of workers studied in 2019 were composed of 68 subjects from the Ischia group and 72 subjects from the Villasimius group with an age ranging between 41 and 50 years. The subjects were free of refraction defects linked to cataract problems and other chronic ocular pathologies such as myopia, their work time of exposition to sunlight was 6-8 hours a day for 6 days a week, the professional categories were fishermen, boatmen, mooring workers and lifeguards [**5**-**6**]. By means of a Snellen optotype, a refraction test was performed on all these workers, and only those who had a natural visus for at least 5/ 10 per eye were enrolled in this study. A portable slit-lamp biomicroscope was used to examine the anterior segment with a particular attention to the degree of opacity of the crystalline lens due to sclerosis. A classification in three degrees was preferred for the opacities of the crystalline lens: incipient cataract, intumescent cataract and mature cataract, although the latter was never found in the sample examined, as they were relatively young subjects, who also had a relatively easy access to health services, due to living in a European country, even though they reported that they were not periodically examined by a competent doctor, based on Legislative Decree number 81/ 08 and subsequent amendments, consequently to the precariousness and autonomy of their work [**5**-**7**].

## Results

As previously mentioned, no worker examined had such a lens opacity that it could be classified as a mature cataract, even though none of the subjects examined in both studied populations were free from these opacities [**8**]. The problem was limited to incipient and intumescent cataracts. For the cohort of Ischia, 90 eyes of 53 people and 81.81% of the total sample were affected by the incipient cataract, while the cohort of Villasimius included 115 eyes with 61 incipient cataracts and 79.86% of the total sample. The remaining eyes of both studied cohorts were categorized as intumescent cataract. Despite the relatively young age of the sample, these subjects had a higher incidence of progression of crystalline sclerosis than the health statistics for cohorts of the same age, although there were no statistically significant differences between the two groups studied, probably due to the constant exposure to sunlight in a strongly reflecting marine environment. The specific genetic factors on Sardinia island, which favor Mediterranean anemia or other congenital or acquired diseases, as well as on the island of Ischia, where fetal and neonatal malformations appear to be statistically favored, do not seem to be important in the case of the pathogenesis of cataracts [**9**,**10**]. 

## Discussion

According to the Italian National Institute of Statistics (ISTAT), cataracts affect 8.5% of the population in Italy between 70 and 74 years old, 12.4% of the following five years and 17.1% of those over 80 years old, but, in the younger population, there are no reliable data on the matter. According to the World Health Organization (WHO), it is the world’s leading cause of blindness and low vision, although it is almost always reversible [**9**]. According to the latest available data, it is also responsible for 53% of the cases of visual disability (but it is often operable or reversible), mainly concentrated in developing countries, while in many cases, there are no resources to carry out the cataract operation [**10**]. Genetic factors play an important role and the processes of aging of the body also occur in crystalline lens [**11**,**12**]. Among the acquired forms of cataract, we recognized the senile cataract that was 90% of all forms, with the onset usually after the age of 50. Age is the main risk factor, while environmental, metabolic or genetic factors can have a cumulative effect [**13**]. According to its location, senile cataract is divided into cortical, nuclear and posterior subcapsular. Cortical cataract is the most frequent form, it can be isolated or associated with nuclear opacity, can affect the anterior cortex, the posterior cortex or more frequently both. The main cause involved in its formation is a hydro electrolytic imbalance that induces hyper hydration and liquefaction of the lenticular fibers. Cortical opacity is usually wedge-shaped and originates from the periphery of the lens with centripetal direction. This type of cataract generates a reduction in visual acuity of varying magnitude and a loss of contrast sensitivity that causes glare from point and intense light sources more pronounced at night; is also particularly penalizes near vision. Diagnosis is made by slit-lamp biomicroscopy, showing radial cuneiform opacity of generally equatorial origin. These signs are better recognizable in backlighting [**14**,**15**]. Nuclear cataracts occur due to the opacification of the nucleus of the lens by the accumulation of insoluble proteins of high molecular weight with a consequent increase in nuclear density. This phenomenon, called nuclear sclerosis, does not initially involve a reduction in visual capacity, but generates an increase in the refractive index of the nucleus with myopization of the eye, which increases as a result of the evolution of nuclear opacification. The related symptoms are myopia with reduction of visual acuity for distant and more marked in mesopic vision (at sunset), occasionally associated with diplopia or monocular polyopia due to a prismatic effect of different parts of the nucleus. The diagnosis by biomicroscopic examination with a direct beam of light (placing the light at 30° and 45°) shows the loss of transparency of the nucleus with a grey-greenish color in the initial phases (initial cataract) and yellow-brown in advanced phases (intumescent cataract). However, when this is associated with the cortical cataract, it is defined as total [**16**]. The posterior subcapsular cataract usually starts at the posterior pole of the lens in the form of fine granular opacity, with the tendency to propagate towards the periphery, constituting a plaque opacity. This type of cataract is very common in diabetic subjects or after a prolonged treatment with corticosteroids. Impairment of vision is particularly severe because the site of opacity is very close to the nodal point. Consequently, there is a difficulty in the near vision with daytime glare, while in the early stages, night vision is quite good. The development of monocular diplopia is then possible due to localized changes in the refractive index. At the slit lamp examination, posterior subcapsular cataract is highlighted as a dark area by placing the light beam in backlight. In an advanced stage, it appears as a thick calcific-like area [**17**]. Cataracts benefit from a single and exclusively surgical approach. The operability is entrusted to a medical and/ or functional criterion. The first one takes into account the mature state of the cataract and the risk of associated complications, the second one is based on the decline in visual acuity and the consequent implications in daily life. The opacification of the lens can also be acquired, as in the case of traumatic cataracts secondary to penetrating traumas that generate a continuous solution of the capsule, the metabolic cataract, linked to systemic diseases, such as galactosemia, Fabry’s disease, Lowe syndrome (also called oculocerebrorenal syndrome) and Wilson’s disease. A secondary toxic cataract is also possible for the prolonged use of topical corticosteroid drugs or for the systemic use with formation of a posterior subcapsular cataract, miotic anticholinesterases and phenothiazines that cause starry brownish deposits under the anterior capsule. The prevalence of senile cataract associated with visual defect varies from northern Italy to the southern, being higher in the south. The prevalence quotient in the population over 40 years old is between 4.7 and 7.2% in the South, with a maximum increase in prevalence in the population over 70 years old [**18**,**19**]. In this scientific work dealing exclusively with cataracts acquired in a young population and favored by the intense exposure to ultraviolet radiation present in sunlight, we made a classification of the acquired cataracts, according to the topographic criterion, into nuclear, cortical, rear and mixed subcapsular cataracts. According to the density of the opacity of the lens, the cataract is classified into incipient cataract, intumescent cataract, mature cataract, hyper-mature cataract (or morgagnian cataract), and, within the latter, white, brunescens and nigra cataracts are differentiated. However, as it can be observed from the data collected in the present study and described above, no statistically significant factors emerged in the two-year study made on the existence of genetic factors predisposing to cataracts in the populations of southern Sardinia especially and Campania islands, consequent to the exposure to intense summer sunlight in a marine environment, although there is obviously a simple aggravation of sclerosis of the lens itself, when compared to the same age groups not professionally exposed [**18**-**20**].

## Conclusions

The study in question, carried out on four homogeneous populations in terms of visual conditions, age and profession, during the weeks of the year in which the sun’s ultraviolet radiation is most intense, has not statistically demonstrated the existence of genetic factors in the isolated populations of Sardinia and Ischia, capable of playing a role of a real “melting moment”, or rather of an efficient and decisive factor in the pathogenesis of the cataract opacity.
